# From mechanical triggering to metabolic-inflammatory driving: a new paradigm of knee osteoarthritis pathogenesis

**DOI:** 10.3389/fimmu.2026.1833313

**Published:** 2026-05-18

**Authors:** Manli Yan, Xin Zhang, Hao Liu, Qingyang Kang, Jianjiang Li, Baoqing Zhang, Dingkun Lin, Xiang Li

**Affiliations:** 1 The Second Clinical College, Guangzhou University of Chinese Medicine, Guangzhou, Guangdong, China; 2 Department of Knee Osteoarthritis, Guangdong Provincial Hospital of Chinese Medicine, Guangzhou, Guangdong, China; 3 Orthopaedic Hospital, Guangdong Provincial Hospital of Chinese Medicine, Guangzhou, Guangdong, China; 4 Department of Orthopaedic Teaching and Research, Guangdong Provincial Hospital of Chinese Medicine, Guangzhou, Guangdong, China

**Keywords:** knee osteoarthritis, mechanical factors, metabolic mechanisms, metabolomics, personalized treatment, systemic inflammation

## Abstract

**Background:**

Knee osteoarthritis (KOA) is a global public health crisis and a leading cause of disability among middle-aged and elderly populations. Historically characterized as a passive “wear-and-tear” process, the understanding of KOA has undergone a fundamental paradigm shift. While aberrant biomechanical loading remains a primary initiator of joint damage, it is increasingly recognized that systemic metabolic dysfunction and chronic low-grade inflammation act as the critical forces driving sustained disease progression.

**Main body:**

Abnormal mechanical loading is the primary initiator of KOA, inducing chondrocyte micro-injury and ultrastructural extracellular matrix disruption. Mechanotransduction via integrins and ion channels activates pro-inflammatory and degradative pathways, such as NF-κB and Wnt/β-catenin, tilting the joint toward a catabolic state. While traditional views emphasize physical attrition, emerging evidence suggests that mechanical stress may be perpetuated by systemic metabolic factors. Obesity potentially acts as a pathophysiological bridge, where the infrapatellar fat pad (IFP) might function as a metabolic hub, possibly translating endocrine signals into local joint inflammation through adipokine secretion and extracellular vesicle communication. Furthermore, molecular investigations into the lipid metabolism axis, including the CH25H-CYP7B1-RORα signaling pathway, provide preliminary evidence for metabolic influence on chondrocyte senescence. While clinical evidence for these metabolic drivers is still evolving, synovial fluid metabolomics is being actively explored to identify potential biomarkers.

**Conclusions:**

The evolving pathological landscape of KOA necessitates a transition in clinical management from isolated symptomatic relief toward integrated metabolic regulatory strategies. This new paradigm—defined by “mechanical initiation, metabolic-inflammatory driving, and heightened mechanical sensitivity”—provides a rigorous theoretical foundation for developing innovative, disease-modifying prevention and therapeutic models, including targeted nutritional interventions and metabolic-modulating pharmacology.

## 1 Introduction

Knee osteoarthritis (KOA) is defined by a characteristic pathological triad: the progressive degradation of articular cartilage, significant subchondral bone remodeling, and chronic synovial inflammation (1, 2). As a pervasive degenerative disorder, KOA represents a global public health crisis; estimates indicate that in 2019, approximately 364.6 million individuals were affected worldwide, accounting for 4.9% of the global burden of disease (3). Beyond its high prevalence, KOA has emerged as a predominant cause of disability and a staggering socioeconomic burden among middle-aged and elderly populations, necessitating a more profound understanding of its multifaceted pathogenesis.

For decades, the prevailing “wear-and-tear” theory has characterized KOA as a passive degenerative process driven by biomechanical loading. Within this framework, mechanical imbalances—such as muscle atrophy and tibiofemoral malalignment—were identified as the primary culprits behind physical cartilage attrition and functional failure (2, 4, 5). However, attributing the complexity of KOA to isolated mechanical friction is increasingly recognized as insufficient. While mechanical insults trigger initial tissue damage (6, 7), systemic metabolic dysregulation has emerged as the critical force driving sustained disease progression. Current evidence advocates that KOA is a degenerative arthropathy orchestrated by the synergistic effects of dysregulated lipid metabolism, low-grade systemic inflammation, and dietary influences, all operating atop an aberrant mechanical baseline (8). Consequently, the pathological paradigm of KOA is undergoing a fundamental transformation: shifting from a reductionist “mechanical model” to an integrated framework defined by “mechanical initiation, metabolic-inflammatory driving, and heightened mechanical sensitivity.” (9–11) To establish a precise theoretical baseline, we distinguish “Mechanical Initiation” as the immediate biophysical response and ultrastructural ECM disruption triggered by physical loading. In contrast, “Metabolic-Inflammatory Driving” refers to the subsequent pathological takeover by systemic metabolic cues (e.g., dyslipidemia, adipokine imbalances) that amplify these initial signals and maintain the joint in a chronic catabolic state. This paradigm shift offers a novel lens through which to reinterpret macro-risk factors like obesity, whose impact transcends static loading to encompass a complex biochemical cascade triggered by dysfunctional adipose tissue acting as a potent endocrine organ (6, 12, 13).

In light of these insights, KOA should no longer be viewed as a localized physical erosion, but rather as a systemic disorder initiated by mechanical triggers and perpetuated by metabolic-inflammatory cascades (10), involving active biological responses of the joint tissues (11). This review deconstructs the core mechanisms of metabolic-inflammatory driving and explores the intrinsic crosstalk between systemic metabolic dysfunction and the local joint microenvironment. Ultimately, we propose individualized intervention strategies based on metabolic endotyping, aiming to provide a robust theoretical foundation for developing innovative “disease-modifying” models for KOA prevention and therapy.

## 2 Mechanical initiation of KOA

Abnormal mechanical loading serves as the fundamental initiator in the pathological evolution of KOA. Articular cartilage is characterized by high mechanosensitivity (14), with its phenotypic homeostasis strictly contingent upon mechanical stimuli within physiological ranges. The onset of KOA pathology is governed not only by macro-mechanical environments but also by the precise regulation of stress redistribution within the local microenvironment (15).

The biomechanical homeostasis of the knee joint is predicated on a dynamic equilibrium involving lower-limb mechanical alignment, meniscal load transmission, and dynamic ligamentous stability (16); the failure of any single component triggers a deleterious pathological cascade (17). For instance, anterior cruciate ligament (ACL) deficiency induces aberrant anterior tibial translation and rotational instability, subjecting the meniscus to shear stresses that exceed physiological limits. The medial meniscus, serving as a secondary constraint for anterior tibial displacement, further exacerbates joint instability upon functional loss (16). Clinical evidence demonstrates a significant positive correlation between medial meniscal extrusion under weight-bearing conditions and increased knee varus thrust. As a hallmark of dynamic malalignment, varus thrust-induced stress concentration in the medial compartment (18, 19) has been identified as the primary physical driver mediating subchondral bone sclerosis and structural progression (19, 20).

Crucially, aberrant biomechanical loading is not merely a process of passive physical attrition, but acts as an initial trigger signal that first induces chondrocyte micro-injury and ultrastructural disruption of the extracellular matrix (21). The cumulative effect of these micro-insults disrupts the joint’s mechano-biological homeostasis, activating aberrant repair phenotypes in chondrocytes. This mechanically-initiated pathological landscape not only compromises the physical barrier of the joint but also facilitates the release of matrix degradation products, thereby establishing the critical pathological groundwork for the subsequent intervention of systemic metabolic dysregulation and inflammatory cascades (22, 23). It must be emphasized that the impact of mechanical loading on joints is dual-natured. Moderate exercise within the physiological range has been demonstrated to exert significant protective effects, delaying the progression of KOA by maintaining chondrocyte metabolic homeostasis, inducing the expression of anti-inflammatory factors, and inhibiting synovial inflammation (24–27). Consequently, the pathological initiation of KOA does not stem from physical activity itself, but rather from a pathological cascade triggered by abnormal mechanical stress that exceeds the physiological compensatory threshold (27, 28).

## 3 Molecular mechanotransduction in KOA

The sophisticated ultrastructure of articular cartilage confers exceptional compressive resistance; however, its inherently limited self-repair capacity renders it highly vulnerable to pathological stress. Research indicates that while physiological mechanical stimulation is indispensable for maintaining the homeostatic balance between chondrocyte anabolism and catabolism (29, 30), excessive or aberrant loading tilts this equilibrium toward a catabolic state, acting as a primary trigger for KOA.

At the molecular level, abnormal mechanical stress is transduced into biochemical signals via mechanoreceptors on the chondrocyte membrane, such as integrins and ion channels (30, 31). This mechanotransduction directly activates multiple pro-inflammatory and degradative signaling pathways, including NF-κB and Wnt/β-catenin. The activation of these pathways subsequently induces the release of pro-inflammatory cytokines, notably interleukin-1β (IL-1β), and significantly upregulates the expression of proteolytic enzymes, such as matrix metalloproteinases (MMPs) and a disintegrin and metalloproteinase with thrombospondin motifs (ADAMTS). Persistent NF-κB signaling serves as a critical driver of chondrocyte senescence, promoting the acquisition of a senescence-associated secretory phenotype (SASP). Through this phenotype, senescent chondrocytes propagate inflammatory signals by altering the state of neighboring cells, remodeling the extracellular matrix, and triggering immune responses, ultimately compromising the intrinsic repair capacity of cartilage (32, 33). Similarly, precise regulation of the Wnt/β-catenin signaling pathway is essential for maintaining cartilage homeostasis, whereas its aberrant activation disrupts the metabolic equilibrium of chondrocytes. Animal studies have demonstrated that inhibition of abnormal β-catenin expression significantly reduces the levels of MMPs and ADAMTS-5, while effectively alleviating pathological damage to articular cartilage (34).

The mechano-induced inflammatory cascade, hypercatabolism, and chondrocyte senescence collectively constitute the pathological core of early-stage KOA, a process further modulated by obesity, genetics, trauma, and metabolic syndrome (35, 36). Of note, this pathological evolution is not confined to the cartilage layer alone. Early studies have emphasized that age-related and pathological progression-induced subchondral bone sclerosis, accompanied by reduced vascularization, substantially impairs the nutritional diffusion capacity of articular cartilage, thereby initiating and accelerating cartilage degeneration (37, 38). Such microenvironmental deterioration exacerbates the survival stress on chondrocytes, further perpetuating the degenerative process.

On this basis, persistent mechanical stress triggers deeper levels of organelle stress and dysfunction (39–41). Bioenergetic failure, cumulative oxidative stress, and mitochondria-mediated apoptotic pathways have been identified as pivotal drivers of cartilage degeneration. Substantial evidence further supports the role of mitochondrial dysfunction in sustaining the chronic progression of osteoarthritis.

**Figure 1:**
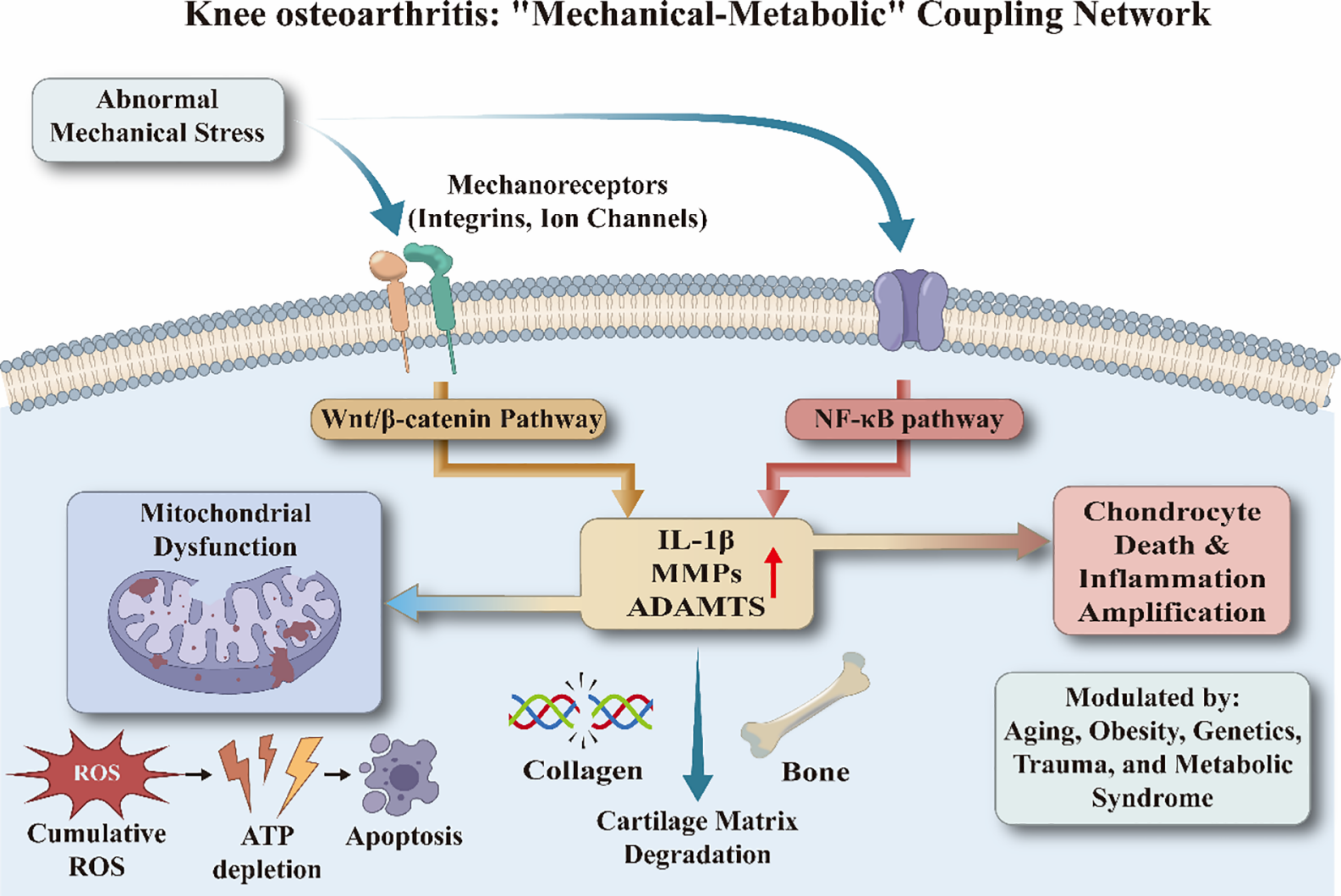
Knee osteoarthritis: “mechanical-metabolic” coupling network.

In summary, abnormal mechanical stress establishes its indispensable initiating role in KOA pathogenesis by inducing micro-injury and activating mechanotransduction pathways. However, the evolution of KOA is not an isolated process of physical attrition; current evidence points toward a more complex “mechanical-metabolic” coupling network (**Figure 1**). Although the precise temporal sequence of metabolic imbalance in KOA remains to be fully elucidated, key metabolites have been shown to profoundly participate in the amplification of inflammatory cascades (42), the disruption of membrane lipid homeostasis, and programmed chondrocyte death. These biological effects—initiated by mechanical stress and perpetuated by metabolic dysregulation—collectively form the core pathological paradigm of sustained KOA progression.

## 4 Obesity and weight intervention in KOA

Obesity is a well-recognized modifiable risk factor for KOA (43). As a safe and effective non-pharmacological intervention, weight loss offers definitive benefits in improving clinical symptoms and functional outcomes. An 18-month randomized controlled trial involving 240 overweight or obese KOA patients demonstrated a significant dose-response relationship between the magnitude of weight loss and improvements in various clinical and mechanistic indicators (44), such as the pro-inflammatory cytokine interleukin-6 (IL-6). Compared to modest weight reduction, a sustained loss of 10%–19.9% of baseline body weight yielded more pronounced clinical and biological advantages. Furthermore, synergistic diet and exercise interventions not only directly reduce body mass but also delay disease progression by optimizing load distribution along the mechanical axis of the lower limb (45). When weight loss exceeds 10%, the resultant force on the knee joint decreases significantly, accompanied by an effective reconstruction of the dynamic stability of the peri-articular musculature. Meanwhile, the maintenance of thigh muscle mass has been identified as a critical macro-protective factor against structural deterioration in KOA (46).

Beyond the optimization of macro-biomechanics, the joint-protective effects of exercise rely more heavily on profound molecular regulatory mechanisms. Regular physical activity serves as a biological rheostat that suppresses chondrocyte apoptosis and exosome-mediated inflammatory transmission by activating intracellular signaling pathways, such as SIRT1. Furthermore, it induces systemic anti-inflammatory responses, thereby effectively antagonizing the chronic metabolic inflammation induced by obesity at the molecular level (47, 48). This exercise-driven molecular protection, synergizing with the mechanical unloading benefits of weight loss, collectively constitutes a robust homeostatic shield for the joint (49).

Similarly, the protective effects of weight loss on KOA extend far beyond the reduction of macroscopic mechanical loading; the core mechanism involves a profound restructuring of the systemic and local metabolic-inflammatory environment (50). Obesity systemically disrupts joint homeostasis by altering tissue composition, promoting inflammatory macrophage polarization, and impairing chondrocyte metabolism (12, 51). Animal studies have demonstrated that in a chronic progressive OA model, CCR2^+^ macrophages infiltrating the synovium originate from Ly6C^+high^ monocytes that are rapidly recruited and become resident in the synovial tissue following injury. The number of these cells is significantly elevated in obese animals at both 3 days and 12 weeks post-injury (52). This indicates that obesity synergistically drives the progression of injury-induced OA by modulating molecular, cellular, and structural alterations within the synovial joint, thereby disrupting synovial immune homeostasis.

Metabolic dysregulation thus serves as a core pathological bridge connecting the macroscopic obesity phenotype to local synovial inflammation (51, 53). Longitudinal imaging studies from the Osteoarthritis Initiative (OAI) revealed that sustained weight loss over 48 months significantly delayed the progression of effusion-synovitis (54), with approximately 39% of this protective effect mediated by the reduction in perijoint subcutaneous fat. Importantly, researchers highlighted that signal changes in the infrapatellar fat pad (IFP) on MRI may reflect the metabolic remodeling of adipose tissue rather than a simple resolution of inflammation. This perspective suggests that obesity should no longer be viewed merely as a static physical load, but as an active pathophysiological hub that orchestrates KOA evolution through its endocrine activity at the microscale. Consequently, the research focus has shifted from macroscale weight management to the endocrine functions of adipose tissue. This serves as a pathophysiological bridge connecting the macroscale obesity phenotype to microscale metabolic dysfunction, providing a core breakthrough for unraveling the “mechanical-metabolic” coupling mechanism in KOA.

## 5 The IFP as a metabolic hub in KOA

Clinical and epidemiological evidence supports a “systemic-to-local” pathological logic in KOA. Studies have demonstrated that in KOA patients with metabolic syndrome, adipokine profiles in both systemic circulation and synovial fluid exhibit a highly consistent shift, characterized by elevated plasma leptin and diminished adiponectin levels (55, 56). Notably, this association remains statistically independent of body mass index (BMI) (55). This suggests that under comparable biomechanical loads, an aberrant endocrine environment shaped by systemic metabolic dysfunction can remotely transmit pro-inflammatory and metabolic signals to the joint via the circulatory system. This phenomenon emphasizes that adipose tissue is no longer merely a passive energy reservoir but a highly active endocrine and immunomodulatory organ playing a central role in regulating systemic inflammation and metabolic homeostasis (57).

As a site-specific adipose structure within the knee, the IFP performs biomechanical functions, such as shock absorption and lubrication (58), while profoundly regulating intra-articular homeostasis by secreting a variety of cytokines, adipokines, and extracellular carriers (59). The IFP represents the largest intra-articular fat depot associated with OA (60), and its size and lipid stores are preserved even under conditions of extreme starvation (61). During the pathological progression of KOA, chemokine-mediated immune cells migrate into the IFP via vascular infiltration, driving a shift in its secretory profile from a homeostatic to a pro-inflammatory phenotype. This transition leads to markedly elevated local levels of inflammatory mediators such as TNF-α and IL-1β, which act in an endocrine, paracrine, and autocrine-like manner (61, 62), thereby initiating and sustaining the cartilage degradation cascade (63, 64). Concurrently, pathological alterations—including neovascularization, fibrotic remodeling, and immune cell aggregation—are prevalent in the IFP tissue of OA patients, with the severity of these changes synchronizing with synovitis and cartilage damage (65). These histological findings suggest that the IFP is not only a source of inflammation in OA progression but a key participant in the reconstruction of the intra-articular immuno-metabolic microenvironment.

Intriguingly, the IFP exhibits a distinct functional duality during the OA process. On one hand, an inflamed IFP continuously releases pro-inflammatory factors, amplifying the local inflammatory cascade. On the other hand, IFP-derived mesenchymal stromal cells possess the potential to differentiate into chondrocytes and promote tissue repair (59). The ultimate biological outcome of the IFP depends on the dynamic equilibrium between inflammatory driving forces and repair potential within the local microenvironment, a balance regulated by systemic metabolic status, immune activation levels, and local mechanical stimuli (60, 66, 67). In essence, the pathological output of the IFP is determined by the net equilibrium between systemic input and local amplification. This bidirectional regulation provides a plausible biological basis for the paradox of persistent inflammation and inadequate repair capacity observed throughout the course of KOA.

A critical focus in recent research is how the IFP precisely transmits metabolic-inflammatory signals to cartilage at the level of intercellular communication to induce phenotypic remodeling. Cutting-edge micro-evidence reveals that the IFP in OA patients can secrete and directionally transport small extracellular vesicles (sEVs) to chondrocytes (58), establishing a direct IFP-cartilage communication axis. In vitro functional studies have confirmed that let-7b-5p and let-7c-5p, which are highly enriched in IFP-derived sEVs, are core effector molecules mediating cartilage injury. By targeting and inhibiting the expression of the lamin B receptor—a negative regulator of senescence—these molecules directly induce a senescent phenotype in chondrocytes and significantly enhance extracellular matrix catabolism. This discovery unveils a role transition of the IFP from an “inflammatory participant” to an “active orchestrator” in OA progression, providing key molecular evidence for how metabolic abnormalities amplify joint degeneration across tissues.

**Figure 2:**
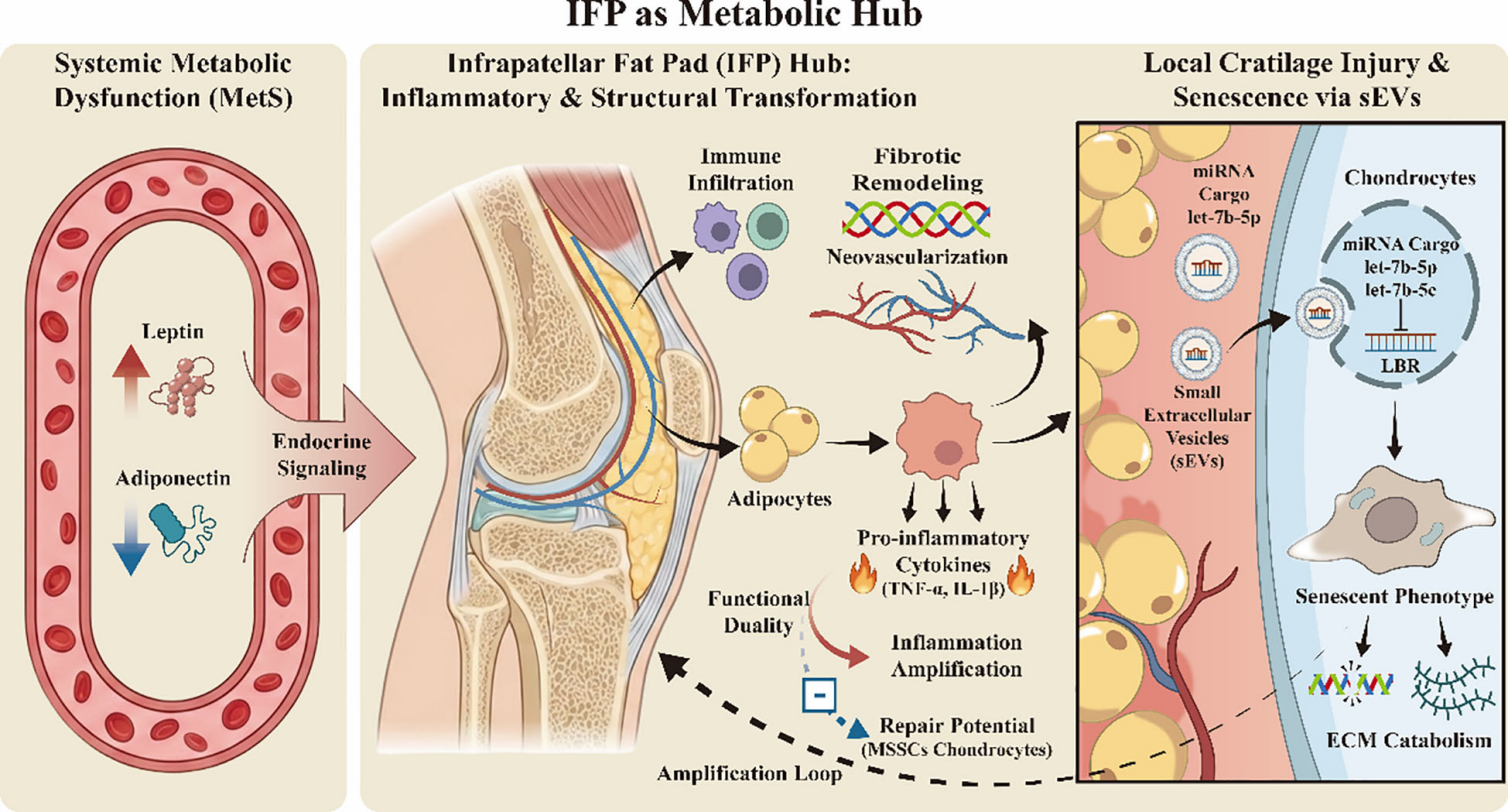
IFP as metabolic hub.

In summary, the IFP is not merely a local reflector of systemic metabolic dysfunction but a vital amplification hub that translates systemic metabolic-inflammatory states into local cartilage injury signals (**Figure 2**). Through a multi-layered mechanism involving inflammatory secretion, structural remodeling, and sEV-mediated molecular communication, the IFP collectively shapes a pro-degenerative microenvironment within the joint cavity. This local amplification effect must synergize with systemic immuno-metabolic imbalances to explain the chronic evolutionary characteristics of sustained KOA progression, establishing a critical foundation for analyzing the comprehensive pathological network of KOA through the “metabolic-inflammatory axis”.

## 6 The metabolic-inflammatory axis in KOA

The aforementioned studies indicate that the IFP can directly translate metabolic abnormalities and inflammatory signals into chondrocyte senescence and matrix catabolism via sophisticated molecular communication mechanisms, such as extracellular vesicles. However, the IFP does not exist in isolation; the formation of its inflammatory and metabolic phenotypes depends on a continuous influx of systemic immunometabolic disturbances. In other words, the amplification effect of degeneration at the local tissue level must be understood within the holistic framework of chronic systemic low-grade inflammation and metabolic imbalance.

Chronic systemic low-grade inflammation is considered the pathological soil driving the sustained progression of KOA. Clinical research has shown that levels of various inflammatory factors in peripheral blood and synovial fluid correlate positively with radiographic severity and clinical symptom scores (68), suggesting that inflammatory load persists throughout all stages of the disease. At the immunometabolic level, age-related metabolic reprogramming of immune cells has emerged as a central focus. Research has found that both the oxygen consumption rate and extracellular acidification rate of B cells in the elderly are simultaneously elevated (69), indicating dual activation of oxidative phosphorylation and glycolytic pathways. This pro-inflammatory metabolic phenotype can induce the polarization of CD4^+^ T cells toward an inflammatory phenotype through lactate-mediated paracrine mechanisms, thereby amplifying the systemic inflammatory cascade. This state of systemic immunometabolic imbalance provides a chronic driving force for persistent local joint inflammation and metabolic remodeling.

Local cartilage metabolic remodeling is a core response to systemic inflammation and environmental stress. As an avascular tissue, articular cartilage metabolism is highly dependent on the diffusive balance of oxygen and nutrients within the synovial fluid (70). Under physiological conditions, chondrocytes maintain energy homeostasis primarily through glycolysis, in which transmembrane transport mediated by glucose transporter 1 (GLUT1) is essential for chondrogenesis and skeletal development (71). Normal chondrocytes possess the compensatory capacity to dynamically regulate GLUT1 expression according to extracellular glucose concentrations (72). While genetic loss of GLUT1 leads to severe chondrodysplasia, pathological overexpression results in excessive glucose uptake and intracellular accumulation of advanced glycation end products (AGEs), thereby accelerating cartilage degradation.

Environmental stressors, such as hypoxia and inflammation, drive characteristic metabolic remodeling in chondrocytes by inducing the abnormal upregulation of GLUT1 (71). During this pathological process, chondrocytes switch from a quiescent state to a highly metabolically active state (73), characterized primarily by a compensatory upregulation of anaerobic glycolysis. Although this metabolic switch aims to address energy crises, the original metabolic homeostasis of the cartilage is completely disrupted under the sustained drive of initial soft tissue injury or abnormal joint loading. Chondrocytes exhibit a distinct immunometabolic shift, leading to a disordered glycolytic cascade and abnormal lactate accumulation, which subsequently triggers significant acidification of the local microenvironment.

Prevention and treatment strategies based on the “metabolic-inflammatory axis” offer new avenues for the individualized treatment of KOA (74). The efficacy of anti-inflammatory diets in improving the clinical manifestations of symptomatic KOA confirms the value of systemic intervention in regulating the local pathological environment (75). Given that KOA exhibits distinct characteristics of systemic metabolic and immune dysfunction, exploring specific serum biomarkers and metabolic phenotypes is of great significance for elucidating its pathogenesis and achieving individualized precision medicine.

## 7 Lipid metabolism and KOA

Serum metabolic biomarkers hold significant prognostic value for risk assessment, disease endotyping, and clinical outcome prediction in knee osteoarthritis KOA (53, 76). Lipid metabolism dysregulation is increasingly recognized as a central manifestation of systemic metabolic dysfunction in KOA (77, 78). For instance, serum levels of apolipoprotein D (ApoD) are markedly downregulated in KOA patients and exhibit a negative correlation with Kellgren-Lawrence grading, VAS scores, and disease duration, while correlating positively with the Hospital for Special Surgery knee score (78). Mechanistic elucidation has confirmed that ApoD exerts chondroprotective effects by activating the PI3K/AKT/mTOR signaling pathway (79). Furthermore, large-scale prospective cohort studies have identified 38 lipoprotein subfractions associated with KOA risk: higher concentrations of extra-large and large high-density lipoprotein (HDL) particles are protective, whereas elevated concentrations of small HDL particles are associated with an increased risk of KOA (80).

Despite the prognostic power of peripheral metabolic profiles, their direct involvement in KOA pathogenesis remains a subject of ongoing debate (81). Due to the avascularity of articular cartilage, systemic serological markers often fail to fully capture the dynamic metabolic fluctuations within the joint microenvironment. Consequently, synovial fluid biomarkers offer superior specificity in reflecting local pathological states. Specifically, synovial levels of HDL-C and apolipoprotein A1 (ApoA1) correlate negatively with cartilage injury scores, radiographic severity, and clinical symptoms (77). Receiver operating characteristic curve analysis has further suggested that HDL-C and ApoA1 as candidate biomarkers for evaluating the severity of advanced KOA. However, translating these candidate biomarkers into clinical practice still faces several challenges, including sample heterogeneity across different cohorts, lack of standardized detection methods, unclear causal relationships, and the invasive nature of synovial fluid collection requiring joint aspiration.

Molecular-level investigations have suggested cholesterol metabolism dysregulation as a potential contributor of KOA pathogenesis (82). The CH25H-CYP7B1-RORα signaling axis has been identified in preclinical studies as a candidate regulator of catabolic metabolism in osteoarthritis (83). In the osteoarthritic state, chondrocytes exhibit enhanced cholesterol uptake, upregulation of cholesterol hydroxylases (CH25H and CYP7B1), and subsequent accumulation of oxysterol metabolites. This leads to pathologically elevated intracellular cholesterol levels, which in turn may trigger cartilage degeneration. However, it should be noted that these findings are primarily derived from animal models; the translational relevance to human KOA requires further validation in clinical settings. Experimental evidence suggests that targeting this pathway—either through genetic ablation, pharmacological inhibition of key enzymes (83), or the promotion of cholesterol efflux (84)—can attenuate OA progression in preclinical models. Additionally, mitochondria-targeted antioxidants and statins have demonstrated potential for cartilage protection by modulating cholesterol metabolic pathways (85).

In summary, the metabolic evolution of KOA represents a sophisticated network integrating systemic and local signals. Relying solely on discrete metabolic biomarkers in serum or synovial fluid is insufficient to fully unveil the complex metabolic dynamics and microenvironmental shifts during disease progression. Driven by advancements in bioanalytical technology, metabolomics has emerged as a transformative tool, enabling the systemic and global assessment of metabolic networks within the joint cavity and throughout the entire organism.

## 8 Synovial fluid metabolomics for KOA endotyping

As a cornerstone of systems biology, metabolomics enables the dynamic quantification of low-molecular-weight metabolites, allowing for the precise capture of subtle pathological fluctuations within the tissue microenvironment. It has emerged as a vital bridge connecting macroscopic clinical phenotypes with microscopic molecular mechanisms. Previous serum-based metabolomic investigations in Southern Chinese populations have identified potential biomarkers such as N-α-acetyl-L-asparagine (86), demonstrating robust predictive capacity for severe KOA, while highlighting the pivotal role of pathways like arachidonic acid metabolism in disease progression. However, metabolomic findings exhibit considerable variability across different study cohorts. This inconsistency primarily arises from differences in geographical regions, dietary habits, racial backgrounds of participants, and standardization of sample processing (87, 88).

In biomarker discovery, urine and serum are frequently utilized due to their accessibility (89). However, metabolites originating from intra-articular lesions must traverse the synovial barrier to enter the peripheral circulation, which potentially dilutes their specificity as local pathological signatures. It is imperative to emphasize that synovial fluid is not merely a simple ultrafiltrate of plasma; its composition is governed by the dual influences of systemic metabolism and the active metabolic contributions of local joint tissues. Consequently, synovial fluid metabolomics offers a more direct and specific reflection of local pathological characteristics.

Previous studies suggest that the IFP and the synovium function as an anatomical-functional unit with the potential to release diverse inflammatory mediators and induce local inflammatory responses (90). Integrated proteomic and lipidomic analyses have identified 315 differentially expressed proteins (DEPs) and eight differential lipid metabolites in the IFP of KOA patients (91). Specifically, levels of ceramide (Cer) and hexosylceramide (HexCer) were downregulated, while six other species (e.g., AcCa, various species of LPC) were upregulated. Enrichment analysis indicated that these DEPs are primarily involved in the complement and coagulation cascades, fatty acid metabolism, and adipogenesis, all of which are closely linked to the immunological dysregulation of the IFP. These findings unveil a specific molecular signature of the IFP at the protein and lipid levels, providing a novel molecular basis for elucidating OA pathogenesis.

Furthermore, integrated metabolomic analyses of synovial tissue and synovial fluid have explored the correlation between metabolic profiles and the severity of synovitis (92). In a cohort involving 37 KOA patients, 42 and 29 metabolites were identified in synovial tissue and fluid, respectively, with only lactate, dimethylamine, and creatine showing significant positive correlations between the two compartments. Further analysis revealed that concentrations of various metabolites (e.g., lactate, alanine, glutamine, and leucine) in synovial tissue were closely associated with synovitis scores. Concurrently, levels of IL-6, acetoacetate, and tyrosine in synovial fluid demonstrated high predictive value for the inflammatory status of the synovial tissue.

**Figure 3:**
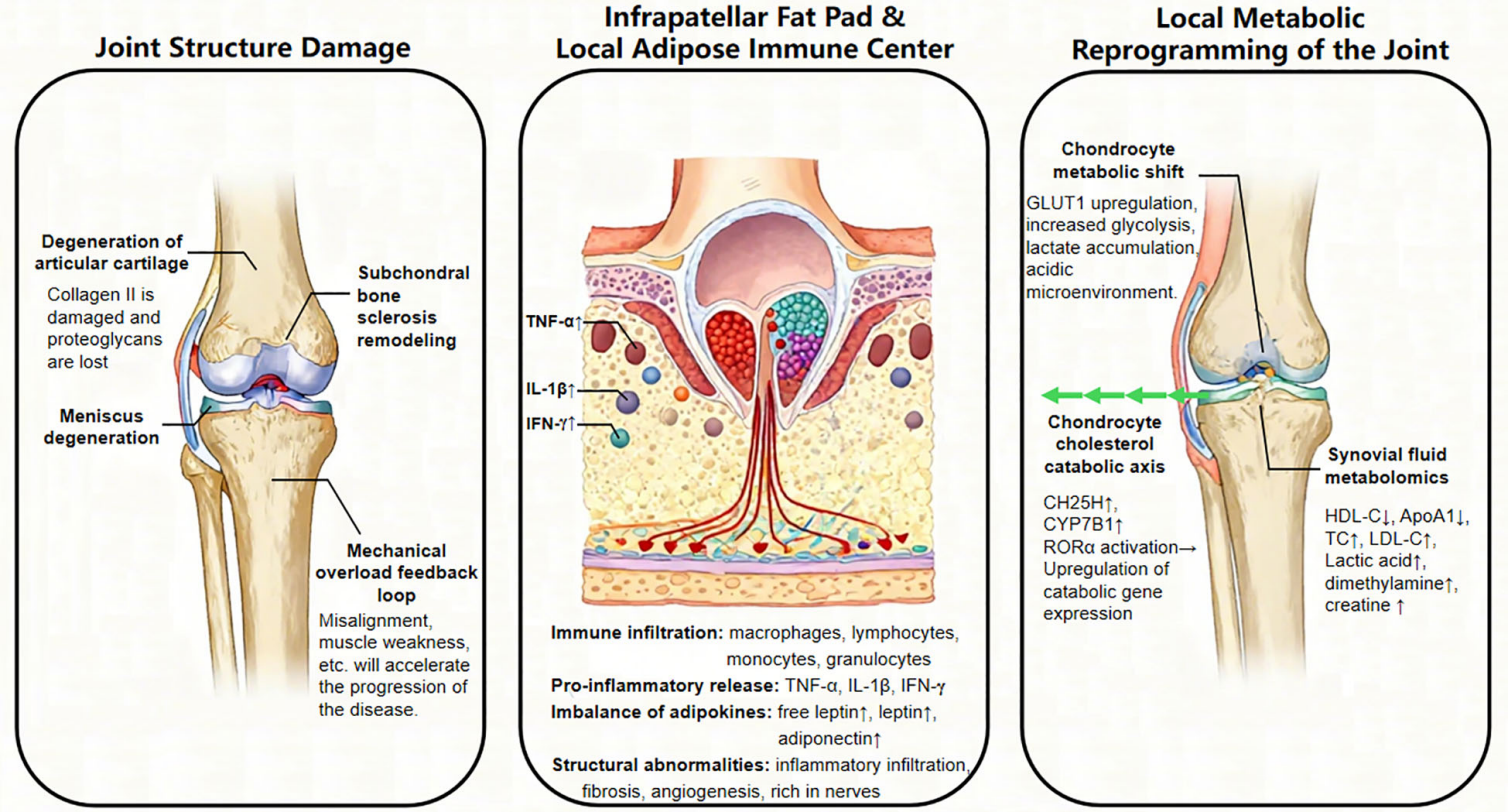
Pathological mechanisms of KOA: transitioning from the traditional “mechanical model” to the new paradigm of “mechanical initiation, metabolic-inflammatory driving, and heightened mechanical sensitivity”.

In summary, the conceptualization of KOA pathogenesis has evolved from a traditional “mechanical model” to a multidimensional paradigm encompassing “mechanical initiation, metabolic-inflammatory driving, and heightened mechanical sensitivity” (**Figure 3**). Under this framework, metabolic derangement within the local joint microenvironment is not merely a pathological byproduct of tissue degradation; rather, it actively alters the physical properties of the extracellular matrix (e.g., matrix stiffness) via biochemical signal transduction. This, in turn, increases the sensitivity of joint tissues to aberrant mechanical loading, establishing a “metabolic-stress” vicious cycle. However, KOA metabolomics remains in an explorative stage. Constrained by limited sample sizes and the intricate crosstalk between systemic and local metabolism, the vast clinical translational potential of this field warrants further in-depth investigation.

## 9 Pain mechanism in KOA

The “heightened mechanical sensitivity” of joint tissues, driven by metabolic-inflammatory remodeling of the extracellular matrix, creates a peripheral environment primed for aberrant nociceptive signaling. The pain mechanism in KOA is not merely a consequence of physical signals arising from cartilage wear; rather, it represents a complex evolutionary process initiated by mechanical stress, sustained by metabolic inflammation, and ultimately culminating in functional remodeling of the nervous system (93). Aberrant innervation occurs throughout all stages of OA pathology and serves as a core driver of pain persistence and chronification (38). KOA pain encompasses both nociceptive and neuropathic components, and may be accompanied by peripheral and/or central sensitization. Regions of synovitis and subchondral bone lesions act as concentrated sites for pro-inflammatory mediator expression. Cytokines such as IL-6, IL-1β, and TNF-α released from these sites not only drive further degeneration of joint tissues but also enhance the responsiveness of nociceptors, thereby shifting the local microenvironment toward a hypersensitive state (94).

Electrophysiological studies using a monoiodoacetate-induced OA model have revealed the dynamic migration pattern of pain signals as the disease progresses. During the early stage characterized by overt inflammation, pain is primarily mediated by the medial articular nerve innervating periarticular tissues such as the joint capsule, manifesting as increased spontaneous activity and reduced mechanical activation thresholds, whereas bone nociceptor function remains largely unchanged at this stage. However, as the disease advances to the late stage, where cartilage erosion involves the subchondral bone, substantial alterations in bone nerve function occur, marked by a pronounced decrease in mechanical activation thresholds and a surge in firing frequency of Aδ and C fibers (95). This transition from joint capsule inflammation to deep bone nociception clearly delineates the biological paradigm of KOA pain evolution from peripheral inflammatory irritation to a deep neuropathic state.

Within the “mechanical-metabolic” coupling network, key metabolites—such as resistin and fatty acid oxidation products—are no longer merely byproducts of tissue damage; instead, they function as neuromodulators that actively participate in pain perception, with local metabolic activity closely correlating with pain intensity (93, 96). Of particular importance, adipose tissue dysfunction is highly implicated in the development of KOA-related pain by inducing local and systemic inflammation, immune dysregulation, and the secretion of pro-inflammatory adipokines. This influence of metabolic disturbance on pain is independent of the biomechanical effects of BMI, thereby constituting a crucial metabolic foundation for pain chronification (97).

As the disease progresses into the chronic stage, sustained input of peripheral nociceptive signals induces functional and structural remodeling of the central nervous system, a phenomenon known as central sensitization (98). Central sensitization is defined as an enhanced responsiveness of nociceptive neurons in the central nervous system to normal or subthreshold afferent input, clinically manifested as hyperalgesia and allodynia. Animal studies have revealed that in the dorsal root ganglia innervating the knee joint, the development of central sensitization most likely originates from peripheral stimulatory input via the L5 segment of the dorsal root ganglia. At the spinal level, the occurrence of secondary hyperalgesia is closely associated with increased expression of TAC1 and NPY (99).

## 10 Conclusion

The pathological understanding of KOA has evolved beyond the traditional confines of “passive mechanical wear” and is now redefined as a complex systemic disorder initiated by mechanical stress and sustained by the synergistic driving forces of systemic metabolic dysfunction and endocrine dysregulation. This emerging paradigm—characterized by “mechanical initiation, metabolic-inflammatory driving, and heightened mechanical sensitivity”—necessitates a fundamental pivot in clinical management: transitioning from isolated symptomatic analgesia toward precision interventions centered on holistic metabolic regulation.

Building upon traditional functional support, the assessment of systemic metabolic status should be integrated into standard clinical pathways. Substantial disease modification may be achieved through customized nutritional interventions, precision exercise prescriptions, metabolic-optimizing pharmacotherapies, and molecular therapies targeting specific metabolic pathways. Furthermore, the integration of high-throughput metabolomics and the discovery of novel biomarkers will facilitate the transformative leap from empirical diagnosis toward metabolic endotype-stratified personalized medicine. Ultimately, this shift provides a robust framework for developing innovative disease-modifying prevention and therapeutic models in KOA management.
